# How do machine learning models perform in the detection of depression, anxiety, and stress among undergraduate students? A systematic review

**DOI:** 10.1590/0102-311XEN029323

**Published:** 2024-12-20

**Authors:** Bruno Luis Schaab, Prisla Ücker Calvetti, Sofia Hoffmann, Gabriela Bertoletti Diaz, Maurício Rech, Sílvio César Cazella, Airton Tetelbom Stein, Helena Maria Tannhauser Barros, Pamela Carvalho da Silva, Caroline Tozzi Reppold

**Affiliations:** 1 Universidade Federal de Ciências da Saúde de Porto Alegre, Porto Alegre, Brasil.

**Keywords:** Students, Machine Learning, Mental Health, Estudantes, Aprendizado de Máquina, Saúde Mental, Estudiantes, Aprendizaje Automático, Salud Mental

## Abstract

Undergraduate students are often impacted by depression, anxiety, and stress. In this context, machine learning may support mental health assessment. Based on the following research question: “How do machine learning models perform in the detection of depression, anxiety, and stress among undergraduate students?”, we aimed to evaluate the performance of these models. PubMed, Embase, PsycINFO, and Web of Science databases were searched, aiming at studies meeting the following criteria: publication in English; targeting undergraduate university students; empirical studies; having been published in a scientific journal; and predicting anxiety, depression, or stress outcomes via machine learning. The certainty of evidence was analyzed using the GRADE. As of January 2024, 2,304 articles were found, and 48 studies met the inclusion criteria. Different types of data were identified, including behavioral, physiological, internet usage, neurocerebral, blood markers, mixed data, as well as demographic and mobility data. Among the 33 studies that provided accuracy assessment, 30 reported values that exceeded 70%. Accuracy in detecting stress ranged from 63% to 100%, anxiety from 53.69% to 97.9%, and depression from 73.5% to 99.1%. Although most models present adequate performance, it should be noted that 47 of them only performed internal validation, which may overstate the performance data. Moreover, the GRADE checklist suggested that the quality of the evidence was very low. These findings indicate that machine learning algorithms hold promise in Public Health; however, it is crucial to scrutinize their practical applicability. Further studies should invest mainly in external validation of the machine learning models.

## Resumo

Os alunos de graduação são frequentemente afetados por depressão, ansiedade e estresse. O aprendizado de máquina pode apoiar a avaliação da saúde mental. Com base na seguinte questão de pesquisa “Qual é o desempenho dos modelos de aprendizado de máquina na detecção de depressão, ansiedade e estresse entre estudantes de graduação?”, objetivou-se avaliar o desempenho desses modelos. As pesquisas foram realizadas no PubMed, Embase, PsycINFO e Web of Science. Foram pesquisados estudos que atendessem aos seguintes critérios: publicados em inglês, estudantes universitários de graduação como população alvo, empíricos, publicados em uma revista científica e que previssem resultados de ansiedade, depressão ou estresse via aprendizado de máquina. A qualidade das evidências foi analisada usando o GRADE. Em janeiro de 2024, foram encontrados 2.304 artigos, e 48 estudos atenderam aos critérios de inclusão. Foram identificados diferentes tipos de dados, incluindo dados comportamentais, fisiológicos, de uso da Internet, neurocerebrais, marcadores sanguíneos, dados mistos, demográficos e de mobilidade. Entre os 33 estudos que forneceram dados de precisão, 30 relataram valores superiores a 70%. A acurácia na detecção de estresse variou de 63% a 100%, ansiedade de 53,69% a 97,9% e depressão de 73,5% a 99,1%. Embora a maioria dos modelos apresente desempenho adequado, deve-se notar que 47 deles realizaram apenas validação interna, o que pode superestimar os dados de desempenho. Além disso, a avaliação GRADE indicou que a qualidade da evidência é muito baixa. Os resultados indicam que os algoritmos de aprendizado de máquina são promissores no campo da Saúde Pública; no entanto, é crucial examinar sua aplicabilidade prática. Estudos futuros devem investir principalmente na validação externa dos modelos de aprendizado de máquina.

## Resumen

Los estudiantes de grado suelen verse afectados por la depresión, la ansiedad y el estrés. El aprendizaje automático puede respaldar la evaluación de la salud mental. Con base en la siguiente pregunta de investigación “¿Cuál es el rendimiento de los modelos de aprendizaje automático en la detección de depresión, ansiedad y estrés entre estudiantes universitarios?”, nuestro objetivo fue evaluar el rendimiento de estos modelos. Se realizaron búsquedas en PubMed, Embase, PsycINFO y Web of Science. Se buscaron estudios que cumplieran con los siguientes criterios: se hubieran publicado en inglés, tuvieran a estudiantes universitarios como población objetivo, fueran empíricos, publicados en una revista científica y que predijeran resultados de ansiedad, depresión o estrés mediante aprendizaje automático. La calidad de las evidencias se analizó mediante GRADE. En enero del 2024 se encontraron 2.304 artículos, y 48 estudios cumplieron con los criterios de inclusión. Se identificaron diferentes tipos de datos, incluidos datos conductuales, fisiológicos, de uso de internet, neurocerebrales, marcadores sanguíneos, datos mixtos, demográficos y de movilidad. Entre los 33 estudios que proporcionaron datos de precisión, 30 reportaron valores superiores al 70%. La precisión en la detección del estrés osciló entre el 63% y el 100%, la ansiedad del 53,69% al 97,9% y la depresión del 73,5% al 99,1%. Aunque la mayoría de los modelos presenta un rendimiento adecuado, cabe señalar que 47 de ellos realizaron únicamente validación interna, lo que puede sobrestimar los datos de rendimiento. Además, la evaluación GRADE indicó que la calidad de la evidencia es muy baja. Los resultados indican que los algoritmos de aprendizaje automático son prometedores en el campo de la Salud Pública; sin embargo, es crucial examinar su aplicabilidad práctica. Los estudios futuros deberían invertir principalmente en la validación externa de los modelos de aprendizaje automático.

## Introduction

University students, such as undergraduate students, are widely affected by mental disorders and psychopathological symptoms, particularly those linked to depressive moods, anxiety, stress, and drug addiction ^1^ ^,^ ^2^. Among university students, 12% to 46% experience some impairment in mental health in the first academic year ^3^. The most recent survey by the World Health Organization (WHO) on university students’ mental health, which included eight countries and approximately 14,000 participants, indicated that approximately 35% of participants presented mental health impairments related to mood (depressive or maniac), anxiety, and drug use, with anxiety being the most prominent ^3^. These mental health impairments have worsened since the emergence of the COVID-19 pandemic ^4^ ^,^ ^5^ ^,^ ^6^.

Several psychosocial stressors are associated with mental health problems such as pressure related to successful academic results, separation from family, and peer relationship problems. In addition, mental health disorders are linked to university dropout ^7^, drug use ^8^, self-harm ^9^, and in more severe cases, suicidal ideation and suicide ^10^. Thus, the accurate detection of these disorders and symptoms can facilitate psychotherapeutic interventions, such as psychotherapies and pharmacological interventions, for preventing mental health problems and harmful psychopathological symptoms.

The detection of these symptoms and disorders is supported by psychological testing, which is a part of psychological assessment. Traditionally, psychological testing has been divided into psychometric self-report instruments and projective tests. Psychometric self-report tests measure psychological constructs ^11^, whereas projective tests use the projection method to estimate psychological characteristics, such as personality and even psychopathological symptoms ^12^, such as the Rorschach test and the House-Tree-Person (HTP) test. However, both methods show certain limitations. Psychometric self-report instruments have measurement errors, are answered considering social desirability, and may even be time-consuming. Projective tests are frequently criticized for issues related to their scientific validity and reliability ^12^.

Machine learning algorithms have been established to provide real-time and accurate predictions and diagnoses and expending less time. machine learning is an intelligent system that debugs itself as it receives feedback to improve its predictive and classifying abilities ^13^. machine learning involves the interaction of several fields such as Artificial Intelligence (AI), Computer Science, and Statistics ^14^. These predictions and classifications may involve variables with linear and nonlinear relationships, and unusual predictors may be used ^15^.

The increasing use of machine learning in psychological assessments has been observed on different fronts. For example, it has been used for the assessment of psychopathological variables, such as depression, anxiety, and stress ^16^ ^,^ ^17^ ^,^ ^18^, personality evaluation ^19^, and positive psychological constructs, such as subjective well-being ^20^. Different systematic reviews on the subject have indicated the potential of evaluating psychological constructs and mental disorders via machine learning ^21^ ^,^ ^22^ ^,^ ^23^ ^,^ ^24^ ^,^ ^25^.

Thus, machine learning may be a promising tool for evaluating psychopathological symptoms in undergraduate students. Despite the systematic reviews that focused on machine learning for mental disorders and psychopathological symptoms such as stress, anxiety, and depression among the general population ^21^ ^,^ ^22^ ^,^ ^23^ ^,^ ^24^ ^,^ ^25^, to the best of our knowledge, no review has focused on measuring psychological machine learning constructs among undergraduate students. Therefore, this systematic review aims to evaluate the performance of machine learning models in predicting and detecting depression, anxiety, and stress among undergraduate university students.

## Method

This systematic review followed the reporting guidelines established by the Preferred Reporting Items for Systematic Reviews and Meta-Analyses (PRISMA) for diagnostic test accuracy ^26^. The research protocol was registered on the International Prospective Register of Systematic Reviews (PROSPERO) platform (registration n. CRD42022232335). All studies included in this systematic review were retrieved in January 2024.

### Search strategy

The research question was: “How do machine learning models perform in the detection of depression, anxiety, and stress among undergraduate students?”. The search strategy was implemented by creating three strings using the population, intervention, comparison, and outcome (PICO) framework. First, the word “students” and correlates were used for the target population of undergraduate students (1). The expression “machine learning” was used for the intervention (in this study, the diagnostic method) (2). Finally, the descriptors of depression, anxiety, and stress were used for the outcomes (3).

The combination of these descriptors generated the following general search strategy: (depression OR anxiety OR stress OR mental health) AND (machine learning OR artificial intelligence OR supervised learning OR unsupervised learning OR big data OR transfer learning OR machine intelligence) AND (students OR college students OR university students), which was applied to the consulted databases. The full search strategy combined natural language terms with controlled vocabulary terms (e.g., MeSH Terms, APA Thesaurus, and Emtree) from the consulted databases in titles and abstracts sections. The full search strategy for each of the databases is presented in Supplementary Material (Box S1; https://cadernos.ensp.fiocruz.br/static//arquivo/suppl-e00029323_4593.pdf).

Articles were searched in PubMed, Embase, Web of Science, and PsycINFO databases. Titles and abstracts were screened and made available on Rayyan platform (https://www.rayyan.ai/) ^27^. Then, two independent reviewers (B.L.S. and P.Ü.C.) accepted or rejected the articles following the inclusion and exclusion criteria. A third researcher (S.C.C.) analyzed the reports that generated disagreements. This procedure was supervised by two seniors researchers (C.T.R. and A.T.S.) with experience in systematic review methodology.

### Eligibility criteria

The inclusion criteria for articles were as follows: (a) published in English; (b) targeted undergraduate university students; (c) empirical study; (d) published in a scientific journal; and (e) predicted anxiety, depression, or stress outcomes via machine learning.

All articles included were read thoroughly. Studies that did not meet the eligibility criteria were excluded from the analysis. Subsequently, the data of interest were extracted via a document in DOC format developed exclusively for this study. The variables evaluated included the authors, country of study, sample characteristics, studies designs, type of data, outcome measure, goals, machine learning algorithms, model’s performance, and data about model’s validation.

### Certainty of evidence assessment 

The *Grading of Recommendations Assessment, Development, and Evaluations* (GRADE) was employed for test accuracy studies to assess the certainty of evidence - also called quality of the evidence ^28^ ^,^ ^29^. GRADE assesses the certainty of evidence based on five domains: risk of bias, indirectness, inconsistency, imprecision, and publication bias. GRADE provides a judgment on the certainty of evidence, classifying it as very low, low, moderate, or high. The general evidence assessment considers the “high” classification as baseline, decreasing depending on the judgment of each of the five domains.

To ensure a homogeneous assessment of the certainty of evidence, the studies were categorized based on the performance metrics they reported. Initially, the quality of evidence was evaluated in the 33 studies that provided accuracy data. For those studies that did not report accuracy specifically, sensitivity or specificity scores were considered (5 studies). When neither accuracy nor sensitivity and specificity were available, the evidence was grouped by the area under the curve (AUC) (3 studies) and positive predictive value (PPV) (2 studies). Finally, all remaining studies that did not report any of the aforementioned metrics were integrated (5 studies).

### Quality of machine learning models

To assess the quality of the included articles, the instrument proposed by Ramos-Lima et al. ^23^ was employed after receiving formal authorization. The tool was built to evaluate the quality of machine learning studies, given the lack of applications within this scope, and is under validation. The instrument was used to evaluate nine criteria: (1) sample representativeness (if the study represents target population heterogeneity), (2) control of the confounding variables (if the study controls for potential confounding variables), (3) assessment of the outcome (how the outcome variable was assessed), (4) use of an machine learning technique (if an machine learning technique was mentioned and employed), (5) presentation of performance statistics (if the performance was reported), (6) management of missing data (how missing data were managed), (7) test unseen (separation of data between test and validation), (8) class imbalance (if the authors address the balance of cases), and (9) feature selection (if the authors address feature selection in the dataset).

### Data analysis

The data was organized and presented via a narrative synthesis of the main results. Due to the wide heterogeneity of the studies, it was not possible to perform a meta-analysis.

## Results

### Selection of relevant articles

**Figure 1:**
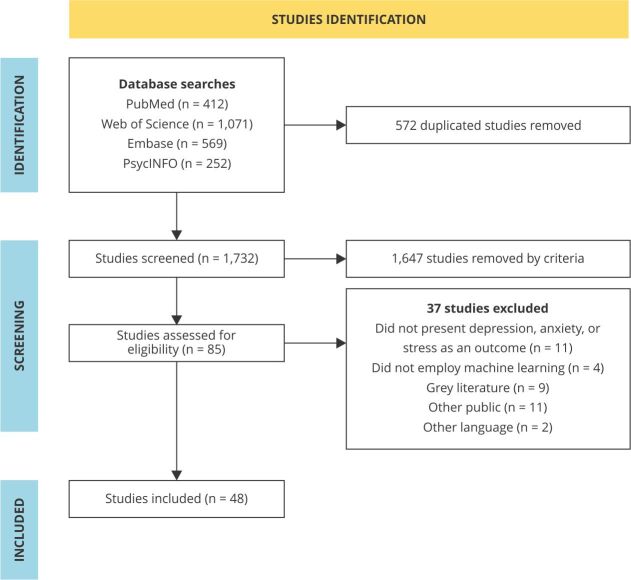
Review process.

After applying the search strategy, 2,304 potential studies, dating from 1988 to 2024, were retrieved from the databases. Of these studies, 412 were from PubMed, 1,071 from Web of Science, 569 from Embase, and 252 from PsycINFO. In total, 85 articles were selected after screening and reading. From these, 48 articles met the inclusion criteria. Figure 1 illustrates the process of selection and exclusion of studies. The list of 37 articles excluded with reasons after full reading is presented in Supplementary Material (Box S2; https://cadernos.ensp.fiocruz.br/static//arquivo/suppl-e00029323_4593.pdf).

### General characteristics of the selected studies

Box 1 outlines the main features of the studies, including the country where it was conducted, the sample size and characteristics, and the study design (e.g., cross-sectional or longitudinal). Most studies were conducted in China (n = 10; 20.83%), European countries (n = 11; 22.91%), or the United States (n = 8; 16.67%). The sample sizes ranged from 24 to 4,184 participants, with ages typically ranging from 17 to 67 years, and a predominant female majority. In total, 36 studies (75%) employed a cross-sectional design.

**Box 1:** Characteristics of the studies.

STUDY (YEAR)	COUNTRY	SAMPLE SIZE	SAMPLE CHARACTERISTICS	STUDY DESIGN
Amalraj et al. ^30^ (2023)	India	24	Not reported	Cross-sectional
Jiao et al. ^31^ (2023)	China	65	Not reported	Cross-sectional
Pal et al. ^32^ (2023)	China	66	Not reported	Experimental
Pourmohammadi & Maleki ^33^ (2020)	Iran	34	67.64% female; 20-37 years (M = 25.4; SD: 4.2)	Cross-sectional
Sharma et al. ^34^ (2022)	India	38	52.6% female; 8-25 years (M = 22.4; SD: 2.42)	Cross-sectional
Silva et al. ^35^ (2020)	Portugal	83	87.95% female; 17-38 years (M = 22.13; SD: 5.5)	Cross-sectional
Tiwari & Agarwal ^36^ (2021)	India	34	Not reported	Cross-sectional
Anand et al. ^37^ (2023)	Saudi Arabia	197	Not reported	Cross-sectional
Balli et al. ^38^ (2023)	Turkey	79	Not reported	Cross-sectional
Daza et al. ^39^ (2023)	Peru	284	Not reported	Cross-sectional
Estabragh et al. ^40^ (2013)	Iran	438	50.22% female (M = 21.37; SD: 2.43)	Cross-sectional
Herbert et al. ^41^ (2021)	Egypt/Germany	220	50.9% female; 18-33 years (M = 20.45; SD: 1.88)	Cross-sectional
Ge et al. ^42^ (2020)	China	2,009	50.95% female	Cross-sectional
Gil et al. ^43^ (2022)	South Korea	171	Not reported	Cross-sectional
Maitre et al. ^44^ (2023)	Canada	3,878	Not reported	Longitudinal
Morales-Rodríguez et al. ^45^ (2021)	Spain	337	73% female; 18-67 years (M = 33.11; SD: 12.83)	Longitudinal
Ren et al. ^46^ (2021)	China	478	57.1% female	Cross-sectional
Upadhyay et al. ^47^ (2023)	India	137	18-25 years	Longitudinal
Vergaray et al. ^48^ (2022)	Peru	284	Not reported	Cross-sectional
Wang et al. ^49^ (2020)	China	1,172	56% female; 18-22 years	Cross-sectional
AlShorman et al. ^50^ (2022)	Saudi Arabia	182	100% male; 18-23 years	Cross-sectional
He et al. ^51^ (2021)	China	589	Not reported	Longitudinal
Li et al. ^52^ (2015)	China	36	33.33% female	Cross-sectional
Modinos et al. ^53^ (2013)	Greece	34	58.82% female; 17-27 years (M = 20.5; SD: 2.4)	Cross-sectional
Zhang et al. ^54^ (2019)	China	82	54.87% female; 18-26 years	Cross-sectional
Liu et al. ^55^ (2023)	China	523	100% female (M = 18.99; SD: 1.1)	Longitudinal
Topalovic et al. ^56^ (2021)	Serbia	100	40% female (M = 22.8; SD: 2.2)	Cross-sectional
Ding et al. ^57^ (2020)	China	693	Not reported	Longitudinal
Dehghan-Bonari et al. ^58^ (2023)	Iran	Not reported	Not reported	Cross-sectional
Siraji et al. ^59^ (2023)	Bangladesh	444	34.45%; 17-20 years	Cross-sectional
Zhang et al. ^60^ (2020)	United States	49	34.69% female	Longitudinal
Ware et al. ^61^ (2020)	United States	182	76.7% female; 18-25 years	Cross-sectional
Aalbers et al. ^62^ (2023)	Netherlands	224	55.8% female (M = 21.97; SD: 3.04)	Longitudinal
Acikmese & Alptekin ^63^ (2019)	Turkey	48	Not reported	Cross-sectional
Ahmed & Ahmed ^64^ (2023)	Bangladesh	100	13% female; 19-30 years	Cross-sectional
Chikersal et al. ^65^ (2021)	United States	138	Not reported	Longitudinal
Guerrero et al. ^66^ (2023)	Ecuador	120	18-24 years old	Cross-sectional
Mahalingam et al. ^67^ (2023)	Lebanon	329	Not reported	Cross-sectional
Meda et al. ^68^ (2023)	Italy	1,388	71.46% female; 18-30 years	Longitudinal
Nemesure et al. ^69^ (2021)	France	4,184	57.4% female	Cross-sectional
Bhadra & Kumar ^70^ (2024)	France	4,184	57.4% female	Cross-sectional
Rois et al. ^71^ (2021)	Bangladesh	355	57.5% female	Cross-sectional
Sano et al. ^72^ (2018)	United States	201	36% female; 18-25 years	Longitudinal
Ware et al. ^73^ (2022)	United States	59	Not reported	Cross-sectional
Xu et al. ^74^ (2021)	United States	397	61.46% female	Longitudinal
Yue et al. ^75^ (2021)	United States	79	18-25 years	Cross-sectional
Müller et al. ^76^ (2021)	United States	57	45.61% female; 18-45 years	Cross-sectional
Nayan et al. ^77^ (2022)	Bangladesh	2,121	55% female; 21-25 years	Cross-sectional

M: mean; SD: standard deviation.

### Machine learning models and performance

Box 2 presents machine learning models organized according to their employed data types. They were grouped into eight main categories: physiological data, behavioral data, neurocerebral data, blood markers, internet usage data, mixed data, mobility data, and demographic data. For each of these models, the illustration presents their primary goals, machine learning algorithms employed, performance parameters reported, methodology for evaluating the outcomes, and whether the model underwent validation. These data are summarized as follows.

**Box 2:** Machine learning models.

TYPE OF DATA	STUDY (YEAR)	OUTCOME MEASURE	GOAL	ALGORITHM	PERFORMANCE	VALIDATION
Physiological data	Amalraj et al. ^30^ (2023)	Not reported	To classify stress among university students	ANN-GA	Accuracy = 99%	Internal validation
Physiological data	Jiao et al. ^31^ (2023)	Not reported	To classify students with depression and without depression and with stress and without stress	Not reported	Accuracy depression = 95.26% Accuracy stress = 98.46% Accuracy depression vs. stress = 100%	Internal validation
Physiological data	Pal et al. ^32^ (2023)	SAS	To classify anxiety among university students	RF	Accuracy = 80% AUC = 82% PPV = 80% Sensitivity = 80% Specificity = 73%	Internal validation with “leave-one-out” cross-validation
Physiological data	Pourmohammadi & Maleki ^33^ (2020)	STAI	To classify stress among university students	SVM	Accuracy = 100% two levels Accuracy = 97.6% three levels Accuracy = 92.2% four levels	Internal validation with nested 10-fold cross-validation
Physiological data	Sharma et al. ^34^ (2022)	BDI-II	To classify the level of depression among university students	AEN	Accuracy = 95.2% five levels	Internal validation with 10-fold cross-validation
Physiological data	Silva et al. ^35^ (2020)	PSS	To classify stress among university students	NN	Sensitivity = 78.1% Specificity = 74.2%	Internal validation with 10-fold cross validation
				NB	Sensitivity = 62.7% Specificity = 74.2%	
				SVM	Sensitivity = 47.5% Specificity = 82.1%	
				RF	Sensitivity = 74.8% Specificity = 71.2%	
				KNN	Sensitivity = 69% Specificity = 75.6%	
Physiological data	Tiwari & Agarwal ^36^ (2021)	PSS	To classify students’ mental state into four categories: relaxed, stressed, partially stressed, and happy	LR	Accuracy = 83.3%	Internal validation with 10-fold cross-validation
				SVM	Accuracy = 88.3%	
				KNN	Accuracy = 82.4%	
				BAG	Accuracy = 98.4%	
				RF	Accuracy = 97.9%	
				GB	Accuracy = 98.2%	
				ANN	Accuracy = 99.4%	
Behavioral data	Anand et al. ^37^ (2023)	QF	Classify the stress of university students into three categories: highly stressed, manageable stress, and no stress	DT + RF + AdaBoost	Accuracy = 93.48% PPV = 92.99% F1 = 93.14% Sensitivity = 93.30%	Internal validation with 5-fold cross-validation
Behavioral data	Balli et al. ^38^ (2023)	BDI	To detect students with depression and without depression	XGBoost	Accuracy = 89.6%	Not reported
Behavioral data	Daza et al. ^39^ (2023)	GAD-7	To predict anxiety level of university students	KNN	Accuracy = 97.83% Sensitivity = 98.44% Specificity = 99.32% F1 = 97.88%	Internal validation with 10-fold cross-validation
Behavioral data	Estabragh et al. ^40^ (2013)	SPI	To diagnose college students with social anxiety	BN	AUC = 89.8%	Not reported
Behavioral data	Herbert et al. ^41^ (2021)	STAI	To predict trait anxiety among university students	SVR, GBR	RMSE = 0.90 % of RMSE in range = 15.04%	Internal validation with test set
Behavioral data	Ge et al. ^42^ (2020)	GAD-7	To predict university students with anxiety	XGBoost	Accuracy = 97.3% Sensitivity = 97.3% Specificity = 96.3%	Internal validation with 5-fold cross-validation
Behavioral data	Gil et al. ^43^ (2022)	CES-D	To predict the risk of depression in college students	RF	Accuracy = 86.27% PPV = 80.59% Sensitivity = 85.00% Specificity = 87.10% F1 = 82.74% AUC = 86.05%	Internal validation
Behavioral data	Maitre et al. ^44^ (2023)	GAD-7	To investigate the anxiety level of university students	LR	R^2^ = 0.5300	Internal validation with 10-fold cross-validation
				LASSO	R^2^ = 0.5294	
				RF	R^2^ = 0.5383	
				XGBoost	R^2^ = 0.5630	
				CatBoost	R^2^ = 0.5656	
Behavioral data	Morales-Rodríguez et al. ^45^ (2021)	PSS	To predict the stress level of college students	ANN	AUC = 74.8%	Internal validation
Behavioral data	Ren et al. ^46^ (2021)	SAS, PHQ-9	To assess depression and anxiety in university students	LR	Accuracy anxiety = 81.42% AUC anxiety = 88.50% Sensitivity anxiety = 83.21% Specificity anxiety = 80.38% Accuracy depression = 73.5% AUC depression = 80.60% Sensitivity depression = 75.3% Specificity depression = 71.80%	Internal validation with 5-fold cross-validation
Behavioral data	Upadhyay et al. ^47^ (2023)	HDRS and CDRS along with clinician diagnostic	To assess persistent depression disorder among university students	Stacked SVM	Accuracy = 89.4% Sensitivity = 89.92% Specificity = 89.96% PPV = 89.82% F1 = 89.96%	Internal validation
Behavioral data	Vergaray et al. ^48^ (2022)	PHQ-9	To predict depression among college students	SVM	Accuracy = 94.69% Sensitivity = 94.22% PPV = 94.09% F1 = 94.12%	Internal validation with 10-fold cross-validation
Behavioral data	Wang et al. ^49^ (2020)	SAS	To predict the level of stress (normal, mild, moderate, severe) at the beginning of the academic semester and one month after the beginning of the academic semester	XGBoost	Model 1 Accuracy anxiety level = 83.81% Accuracy anxiety change = 79.26% Model 2 Accuracy anxiety level = 82.10% Accuracy anxiety change = 84.38%	Internal validation with test set
Neurocerebral data	AlShorman et al. ^50^ (2022)	DASS-21	To detect mental stress among university students	SVM with RBF kernel	Accuracy = 81.40% AUC = 86.10% F1 = 81.40% PPV = 81.50% Sensitivity = 84.40% Specificity = 81.50%	Internal validation
Neurocerebral data	He et al. ^51^ (2021)	STAI	To classify anxiety in university students in comparison to healthy controls, individuals with depression and individuals with schizophrenia	BLR	Accuracy control vs. anxiety = 68.72% AUC control vs. anxiety = 72% Sensitivity control vs. anxiety = 71.40% Specificity control vs. anxiety = 65% Accuracy major depression vs. anxiety = 53.68% AUC major depression vs. anxiety = 53% Sensitivity major depression vs. anxiety = 72.20% Specificity major depression vs. anxiety = 33.13% Accuracy schizophrenia vs. anxiety = 59.1% AUC schizophrenia vs. anxiety = 59% Sensitivity schizophrenia vs. anxiety = 32.88% Specificity schizophrenia vs. anxiety = 73.91%	Internal validation with 10-fold cross-validation and external validation
Neurocerebral data	Li et al. ^52^ (2015)	BDI-II	To classify students with depression and without depression	KNN	Accuracy = 99.1% AUC = 99.9%	Internal validation with 10-fold cross-validation
Neurocerebral data	Modinos et al. ^53^ (2013)	BDI-II	To classify depression among university student	SVM	Accuracy = 77% Sensitivity = 71% Specificity = 82%	Internal validation with “leave-one-out” cross-validation
Neurocerebral data	Zhang et al. ^54^ (2019)	TAS along with clinician diagnostic	To classify students with high anxiety and low anxiety	CNN	Accuracy = 86.5% PPV = 84% Sensitivity= 100% F1 = 91.1%	Internal validation with 5-fold cross-validation
Blood marker data	Liu et al. ^55^ (2023)	CES-D	To predict depression among university women over a 1-year period	SVM	R = 0.81; p < 0.001	Internal validation with test set
Blood marker data	Topalovic et al. ^56^ (2021)	DASS-21	To predict the increase in stress levels among university students	BLR	Accuracy = 70% Nagelkerke R^2^ = 0.38 Snell R^2^ = 0.28	Not reported
Internet dada	Ding et al. ^57^ (2020)	QF	To classify depression among university students	RBF-NN	Accuracy = 82%	Internal validation with test set
				SVM	Accuracy = 80%	
				KNN	Accuracy = 79%	
				DISVM	Accuracy = 86%	
Internet data	Dehghan-Bonari et al. ^58^ (2023)	Not reported	To diagnose students with and without depression	RF	Accuracy = 94%	Internal validation
Internet data	Siraji et al. ^59^ (2023)	DASS-21	To detect college students with and without depression	SVM	Accuracy = 85.14% F1 = 84.92% AUC = 98.41%	Internal validation with 5-fold cross-validation
Internet data	Zhang et al. ^60^ (2020)	PHQ-9, GAD-7	To predict deterioration of depression and anxiety among university students	OLS	Depression (MSE = 2.37, R^2^ = 0.84) Anxiety (MSE = 2.48, R^2^ = 0.81)	Internal validation with “leave-one-out” cross-validation
Internet data	Ware et al. ^61^ (2020)	PHQ-9/QIDS along with clinician diagnostic	To predict various symptoms of depression in university students	SVM with RBF kernel	Model 1 F1 = 67% PPV = 71% Sensitivity = 64% Specificity = 73% Model 2 F1 = 72% PPV = 65% Sensitivity l = 81% Specificity = 51%	Internal validation with “leave-one-out” cross-validation
Mixed data	Aalbers et al. ^62^ (2023)	SESS	To assess the stress of university students	LASSO	MAE = 0.84	Internal validation with 10-fold cross-validation
				SVM	MAE = 0.84	
				RF	MAE = 0.84	
Mixed data	Acikmese & Alptekin ^63^ (2019)	QF	To classify university students into stressed and non-stressed	LSTM	Accuracy = 63% PPV = 63% Sensitivity = 63% F1 = 63%	Internal validation with test set
Mixed data	Ahmed & Ahmed ^64^ (2023)	PHQ-9	To identify depressed and non-depressed students	BFS	Accuracy = 78% PPV = 77.4% AUC = 78% Specificity = 75.50% Sensitivity = 80.4% F1 = 78.80%	Internal validation with 10-fold cross-validation
Mixed data	Chikersal et al. ^65^ (2021)	BDI-II	To classify depression among university students at the end of the semester, as well as depression worsening	AdaBoost	Accuracy = 85.7% depression end of semester F1 = 82% depression end of semester Accuracy = 88.1% depression worsening F1 = 81% depression worsening	Internal validation with “leave-one-out” cross-validation
Mixed data	Guerrero et al. ^66^ (2023)	AMAS-C	To identify college students with and without anxiety	Not reported	Model 1 (facial expression) PPV = 86.84% Model 2 (emotions recognition) PPV = 84.21%	Internal validation
Mixed data	Mahalingam et al. ^67^ (2023)	BAI	To classify university students with and without anxiety	MLP	AUC = 80.70% Accuracy = 67.5%	Not reported
				LR	AUC = 77.25% Accuracy = 67.67%	
				SVM	AUC = 76.01% Accuracy = 69.70%	
				RF	AUC = 74.75% Accuracy = 67.68%	
				XGBoost	AUC = 72.58% Accuracy = 63.64%	
Mixed data	Meda et al. ^68^ (2023)	BDI-II	To assess the change of depression symptoms in university students after six months	RF	Overall PPV = 77% PPV in depression worsening = 49%	Internal validation
Mixed data	Nemesure et al. ^69^ (2021)	Clinician diagnostic	To classify university students with generalized anxiety disorder and major depressive disorder	XGBoost classifier	Generalized anxiety disorder AUC = 73% Generalized anxiety disorder sensitivity = 70% Generalized anxiety disorder specificity = 66% Major depressive disorder AUC = 67% Major depressive disorder sensitivity = 55% Major depressive disorder specificity = 70%	Internal validation with 5-fold cross-validation
Mixed data	Bhadra & Kumar ^70^ (2024)	Clinician diagnostic	To detect students with and without depression	ANN	Accuracy = 88.46%	Internal validation
				SVM	Accuracy = 88%	
				RF	Accuracy = 88.46%	
				XGBoost	Accuracy= 84.18%	
Mixed data	Rois et al. ^71^ (2021)	QF	To classify stress among university students	AdaBoost	Accuracy = 89%	Internal validation with 10-fold cross-validation
Mixed data	Sano et al. ^72^ (2018)	PSS	To classify the stress of university students into high stress and low stress	SVM with RBF	Accuracy = 81.5% F1 = 83%	Internal validation with “leave-one-out” cross-validation
				SVM linear	Accuracy = 70.3% F1 = 72%	
				LASSO	Accuracy = 67.6% F1 = 74%	
Mixed data	Ware et al. ^73^ (2022)	PHQ-9/QIDS along with clinician diagnostic	To predict depression among college students	SVM	F1 = 82% PPV = 78% Sensitivity = 86% Specificity = 74%	Internal validation
Mixed data	Xu et al. ^74^ (2021)	BDI-II	To detect students with and without depression	Not reported	Accuracy = 79.10% PPV = 81.40% Sensitivity = 85.40% F1 = 83.30%	Internal validation with leave-one-out cross-validation
Mixed data	Yue et al. ^75^ (2021)	PHQ-9 along with clinician diagnostic	To predict depression among college students	SVM with RBF kernel	F1 = 79% PPV = 77% Sensitivity = 79% Specificity = 72%	Internal validation with “leave-one-out” cross-validation
Mobility data	Müller et al. ^76^ (2021)	QF based on ICD-10	To predict depression among college students	RF	AUC = 82%	Internal validation
Demographic data	Nayan et al. ^77^ (2022)	PHQ-9, GAD-7	To predict college students with depression and anxiety	KNN	Accuracy depression = 88.28% Sensitivity depression = 66.67% Specificity depression = 96.13%	Internal validation with 10-fold cross-validation
				RF	Accuracy anxiety = 91.49% Sensitivity anxiety = 67.77% Specificity anxiety = 98.53%	

AdaBoost: Adaptive Boosting; AEN: Autoencoder Network; AMAS-C: *Adult Manifest Anxiety Scale - College*; ANN-GA: Artificial Neural Network with a Genetic Algorithm; ANN: Artificial Neural Network; AUC: area under the curve; BAG: Bootstrap Aggregating (Bagging); BAI: *Beck Anxiety Inventory*; BDI-II: *Beck Depression Inventory - Second Edition*; BFS: Boruta Feature Selection; BLR: Bayesian logistic regression; BN: Bayesian Network; CDRS: *Cornell Dysthymia Rating Scale*; CES-D: *Center for Epidemiologic Studies Depression* scale; CNN: Convolutional Neural Network; DASS-21: *Depression, Anxiety, and Stress Scale - 21 Items*; DISVM: Deep Integrated Support Vector Machine; DT: Decision Tree; GAD-7: *Generalized Anxiety Disorder 7*; GB: Gradient Boosting; GBR: Gradient Boosting regression; HDRS: *Hamilton Depression Rating Scale*; ICD-10: *International Classification of Diseases - 10th revision*; KNN: k-Nearest Neighbors; LASSO: Least Absolute Shrinkage and Selection Operator; LR: logistic regression; LSTM: Long Short-Term Memory; MLP: multi-layer perceptron; MAE: mean absolute error; MSE: mean square error; NB: Naive Bayes; NN: Neural Network; OLS: ordinary least squares; PHQ-9: *Patient Health Questionnaire-9*; PPV: positive predictive value; PSS: *Perceived Stress Scale*; QF: qualitative feedback; QIDS: *Quick Inventory of Depressive Symptomatology*; RBF: radial basis function; RBF-NN: Radial Basis Function Neural Network; RF: Random Forest; RMSE: root mean square error; SAS: *Self-Rating Anxiety Scale*; SESS: *Stress Experience Sampling Scale*; SPI: *Social Phobia Inventory*; STAI: *State-Trait Anxiety Inventory*; SVM: Support Vector Machine; SVR: Support Vector Regression; TAS: *Test Anxiety Scale*; XGBoost: Extreme Gradient Boosting.

### Models employing physiological data

This subsection encompasses seven distinct machine learning models exclusively employing physiological data. These data encompass parameters such as breathing, skin conductance, skin temperature, blood pressure, heart rate, and another physiological signal derived from electrocardiograms, electromyograms, and electroencephalograms (EEG).

Amalraj et al. ^30^ used physiological data such as body temperature, skin conductance, sweat rate, sweat pH, and acceleration to evaluate different levels of stress among university students (high stress, medium stress, and low stress). The Artificial Neural Network (ANN) with a genetic algorithm achieved a 99% accuracy rate in detecting stress levels.

Jiao et al. ^31^ employed pulse rate variability metrics to detect depression and stress among university students. They achieved a 95.26% accuracy in detecting depression and 98.46% in detecting stress.

Pal et al. ^32^ aimed to classify students with and without anxiety considering information from cardiac signals. Their Random Forest (RF) algorithm achieved an accuracy of 80%.

Pourmohammadi & Maleki ^33^ aimed to classify stress levels in university students by combining physiological signals from electrocardiograms and electromyograms. Stress was induced in the laboratory via experiments such as the Stroop color and word test and mental arithmetic. The study employed a Support Vector Machine (SVM) algorithm, achieving a stress classification 100% accuracy for two levels, 97.6% for three levels, and 96.2% for four levels.

Sharma et al. ^34^ used electrodermal data such as skin conductance to identify students with and without depression following an experiment involving sound stimuli to evoke emotions. They achieved an accuracy of 95.2% using the Autoencoder Neural Network.

Silva et al. ^35^ sought to predict stress in university students based on heart rate and heart rate variability data. The Neural Network (NN) algorithm exhibited the best performance, with a specificity of 74.2% and a sensitivity of 78.1%.

Tiwari & Agarwal ^36^ developed an machine learning model to assess four distinct mental states: relaxation, stress, partial stress, and happiness. Data sources included parameters such as skin conductivity, heart rate, and blood pressure. These mental states were induced via experimental tasks in the laboratory. The ANN algorithm demonstrated a 99.4% accuracy in detecting these mental states.

### Models employing behavioral data

This subsection encompasses 13 machine learning models constructed from behavioral data obtained via self-report instruments. These models were developed using various data sources, including psychopathological symptoms (e.g., anxiety, paranoia, and anger), personality traits, cognitive beliefs, daily activities, and self-concept information. 

Anand et al. ^37^ assessed various levels of stress (high stress, manageable stress, and no stress) based on students’ behavioral habits during graduation, including sleep duration, productive time, and completion of academic tasks. They employed a combination of Decision Trees (DT), RF, and AdaBoost algorithms, achieving a 93.48% accuracy.

Balli et al. ^38^ developed an algorithm to detect individuals with depression and without depression based on psychopathological symptoms, including variables such as anxiety, stress, and childhood trauma. A 89.6% accuracy was attained using an XGBoost algorithm.

Daza et al. ^39^ developed a model based on anxiety symptoms to predict different levels of anxiety (no anxiety, mild, moderate, or severe). The K-Nearest Neighbors (KNN) algorithm demonstrated a 97.83% accuracy.

Estabragh et al. ^40^ developed an algorithm for assessing social anxiety based on cognitive and behavioral factors, including self-efficacy, attachment patterns, behavioral inhibition, and shyness. The Bayesian Network (BN) algorithm demonstrated an AUC of 89.8%.

Herbert et al. ^41^ evaluated university students’ trait anxiety, measured by the *State-Trait Anxiety Inventory* (STAI), at the outset of the COVID-19 pandemic. They integrated a range of psychological data, encompassing personality traits, mental health indicators, self-concept information, and health beliefs. The Support Vector Regression (SVR) algorithm yielded an root mean square error (RMSE) of 0.90 with 15.4% variation.

Ge et al. ^42^ developed a machine learning model for predicting anxiety in university students. The model was constructed using mental health data, including variables related to suicidal ideation, relationship issues, anxiety levels, and sleeping difficulties. The XGBoost algorithm demonstrated a 97.3% accuracy in predicting anxiety, with a 97.3% sensitivity and a 96.3% specificity.

Gil et al. ^43^ aimed to predict the risk of depression among university students using family and individual behavioral data, including family adaptation and cohesion, family bonds, marital satisfaction, personality, health habits, among others. The RF algorithm achieved a 86.27% accuracy.

Maitre et al. ^44^ explored anxiety level among university students using behavioral data. The CatBoost algorithm yielded an R^2^ value of 0.56.

Morales-Rodríguez et al. ^45^ predicted stress levels using information on the resilience and coping strategies of university students. The ANN algorithm achieved an AUC of 74.8%.

Ren et al. ^46^ aimed to assess the anxiety and depression levels of students during the COVID-19 pandemic using behavioral factors associated with the disease, such as mask-wearing, quarantine status, presence of infected friends, and frequent fever measurements. The RF algorithm achieved a 73.5% accuracy for depression and 81.42% for anxiety.

Upadhyay et al. ^47^ developed a model based on behavioral data to detect persistent depression disorder among university students. The SVM algorithm achieved an accuracy of 89.4%.

Vergaray et al. ^48^ used symptoms of depression to identify students with depression. The SVM algorithm demonstrated a 94.69% accuracy.

Wang et al. ^49^ aimed to assess anxiety levels among university students, measured by the *Self-Rating Anxiety Scale* (SAS), both at the beginning of the academic semester and one month after the commencement of the academic semester, which coincided with the onset of the COVID-19 lockdown. The most effective machine learning model consisted of 20 SAS items and used an XGBoost algorithm, which achieved a 82.1% accuracy in predicting anxiety and a 84.38% accuracy in predicting changes in anxiety levels.

### Models employing neurocerebral data

This subsection encompasses five machine learning models that employed neurocerebral data, including neuroimaging data revealing brain regions activated during specific activities, such as the prefrontal cortex, amygdala, and temporal lobe. AlShorman et al. ^50^ introduced a model for stress classification among university students employing brain EEG signals. Their SVM model with radial basis function (RBF) kernel demonstrated an 81.4% accuracy in stress detection.

He et al. ^51^ developed a machine learning model to assess depression and anxiety in university students. The model employed neuroimages derived from the connectome. The Bayesian logistic regression (BLR) machine learning model achieved a 68.72% accuracy in distinguishing anxious university students from healthy controls and 53.68% accuracy in distinguishing anxiety from depression.

Li et al. ^52^ employed data from the EEG during a free viewing task to differentiate between students with depression and those without. Their KNN algorithm demonstrated a 99.1% accuracy in correctly classifying individuals with depression.

Modinos et al. ^53^ also constructed a machine learning model using neuroimaging data, with the objective of accurately classifying students with and without depression. The SVM algorithm showed a 77% accuracy in classifying depression, along with a 71% sensitivity and 82% specificity.

Zhang et al. ^54^ aimed to accurately identify students with and without anxiety using EEG data acquired during an emotional Stroop test. They achieved an 86.5% accuracy using a Convolutional Neural Network (CNN).

### Models employing blood markers

This subsection discusses two machine learning models that employ data associated with blood markers, including indicators of blood stasis (poor blood circulation or blockage of blood flow in the body) and biomarkers, such as the chromatin of neutrophils in peripheral blood.

Liu et al. ^55^ developed a model based on the constitution of blood stasis to predict depression in female university students, measured by the *Center for Epidemiologic Studies Depression* (CES-D) scale, over a 1-year period. The SVM algorithm was employed. The constitution of blood stasis successfully predicted depression over the course of one year (r = 0.81; p < 0.01).

Topalovic et al. ^56^ constructed a model based on the organization of peripheral blood neutrophils to forecast an increase in stress among university students. The BLR algorithm achieved a 70% accuracy.

### Models employing internet usage data

This subsection covers five machine learning models that were constructed using data sourced from the internet. Examples of these data sources include patterns of social network usage (text interactions and engagement with other users) and browsing activities on web browsers.

Ding et al. ^57^ developed a machine learning model for classifying depression among university students based on user interaction data from a Chinese social network called Sina Weibo (https://weibo.com). This data included elements such as the words used, likes, and emojis. The Deep Integrated Support Vector Machine (DISVM) algorithm showed the best performance, achieving an 86% accuracy in classifying students with depression. 

Dehghan-Bonari et al. ^58^ employed sentiment analysis of texts and interactions on social networks to classify students with severe, moderate, and mild depression. The RF algorithm achieved a 94% accuracy.

Siraji et al. ^59^ aimed to evaluate students with depression using internet connectivity data. The SVM algorithm demonstrated an 85.14% accuracy.

Zhang et al. ^60^ constructed a machine learning model to assess the exacerbation of depression and anxiety in university students during the COVID-19 social isolation. This model was based on search data from Google Search (https://www.google.com/) and YouTube (https://www.youtube.com/) and used an ordinary least square (OLS) algorithm. Temporal aspects of platform usage, including search times, proved to be the most effective predictors of the exacerbation of depression (mean squared error - MSE = 2.37; R^2^ = 0.84) and anxiety (MSE = 2.48; R^2^ = 0.81).

Ware et al. ^61^ developed two machine learning models to assess different depression symptoms, including physical, affective, and cognitive aspects. The models used smartphone usage data, with one based on a local app (Model 1) and the other on data obtained via the wireless network (Model 2). Both models were evaluated using an SVM algorithm with an RBF kernel. Model 1 achieved 67% accuracy in identifying lethargy, whereas Model 2 achieved 72% accuracy in identifying sleep problems.

### Models employing mixed data

In this subsection, we encompass 13 machine learning models constructed using mixed data. Here, models employed some of the previously mentioned data types, such as physiological, psychological, and internet usage patterns, but in conjunction with data not previously discussed, including smartphone activity, geolocation, mobility, among others.

Aalbers et al. ^62^ developed a model based on digital markers such as smartphone login data, messages, and sleep inferences to assess stress among students. The RF algorithm yielded a mean absolute error (MAE) of 0.84.

Acikmese & Alptekin ^63^ employed a machine learning model to classify stress levels in university students, which were assessed via qualitative feedback (indicating whether or not they were feeling stressed). The model primarily relied on smartphone usage data, including light sensor data, audio usage, call conversations, and wi-fi data, as well as geolocation and physical activity. The Long Short-Term Memory (LSTM) algorithm achieved a 63% accuracy in detecting stressed university students.

Ahmed & Ahmed ^64^ assessed students with and without depression using digital marks captured by an app on their smartphones. The BFS algorithm was 78% accurate in identifying students with and without depression.

Chikersal et al. ^65^ developed a model that incorporated geolocation and movement data, as well as smartphone usage patterns, conversations, audio inferences, and contacts. The model aimed to classify students with depression at the end of the academic semester, as well as to predict the worsening of these symptoms. The AdaBoost algorithm successfully identified 85.7% of students with depression at the end of the semester and 88.1% of those with worsening depression symptoms.

Guerrero et al. ^66^ constructed two models to identify students with anxiety: one based on facial expressions (Model 1) and another based on emotional expressions in Facebook (https://www.facebook.com/) posts (Model 2). Model 1 achieved a PPV of 86.84%, whereas Model 2 achieved a PPV of 84.21%.

Mahalingam et al. ^67^ constructed a model employing demographic information including gender, income, and age, as well as health habits such as diet, sleep, and alcohol and cigarette use. The SVM algorithm demonstrated an accuracy of 69.7% in identifying students with anxiety.

Meda et al. ^68^ employed demographic and behavioral data, including income, location, diet, and suicidal ideation, to predict the worsening of depression among university students over six months. The RF algorithm exhibited a PPV of 77%.

Nemesure et al. ^69^ developed a machine learning model using physiological data (such as blood pressure and heart rate), body data (height and weight), psychological data (life satisfaction), and health habits (smoking, diet, physical activity) to classify major depressive disorder and generalized anxiety disorder among university students. The XGBoost algorithm achieved an AUC of 73% in the classification of generalized anxiety disorder and an AUC of 67% in the classification of major depressive disorder. Bhadra & Kumar ^70^ reanalyzed the same dataset and achieved an 88.46% accuracy in detecting depression using a RF algorithm.

Rois et al. ^71^ constructed a machine learning model that integrated physiological metrics, including blood pressure and pulse rate, along with health-related habits data such as body mass index, sleep patterns, and physical activity, for the purpose of categorizing stress levels among university students. The results were assessed based on qualitative feedback from the participants, in which they indicated whether they felt stressed or not. The RF algorithm exhibited an 89% accuracy in stress identification.

Sano et al. ^72^ developed an machine learning model to assess stress in university students. The model was composed of different types of data, such as physiological data (skin conductance and temperature), geolocation data, mobility, cell phone usage patterns (including calls and messages), and social network usage, among others. The SVM with RBF kernel algorithm demonstrated an 81.5% accuracy in classifying university students with high stress and low stress over a period of one month.

Ware et al. ^73^ employed social interaction data from smartphones, including messages and calls, to distinguish between students with and without depression. The XGBoost algorithm achieved an F1 score of 82%.

Xu et al. ^74^ developed a model that incorporated information extracted from cell phone use, such as calls and location data, as well as step and sleep data obtained via a wearable sensor, to detect students with and without depression throughout the academic semester. The developed algorithm demonstrated a 79.1% accuracy.

Yue et al. ^75^ developed a model integrating geographic location data and wi-fi access information from smartphones to detect university students with depression. The SVM with RBF kernel algorithm achieved an F1 score of 79%.

### Models employing mobility data

Only one study used only mobility data. Müller et al. ^76^ classified students with and without depression based on GPS mobility data. The RF algorithm presented an AUC of 82%.

### Models employing demographic data

In a single study focusing on demographic data, Nayan et al. ^77^ aimed to identify students with and without depression, as well as those with and without anxiety by employing variables such as gender, education, professional occupation, and years of study. Their KNN algorithm achieved an accuracy of 88.28% in detecting depression, whereas the RF algorithm demonstrated an accuracy of 91.49% in detecting anxiety.

### Certainty of evidence of the selected studies

The GRADE assessment revealed very low quality of evidence in all studies. Serious risks of bias were found, mainly due to issues in the assessment of outcomes. Furthermore, the indirectness dimension was also scored as serious, given that few studies employed the assessment of clinical professionals in diagnosing outcomes. Additionally, the imprecision dimension was also classified as “serious” since most datasets do not seem to adequately represent the college students population. On the other hand, the inconsistency was considered “not serious,” as the variability of performance scores and instruments used reflect particular characteristics of the studies such as the type of sample, being was already expected ^29^. Finally, no publication bias was identified. Box 3 presents this information in detail.

**Box 3:** Certainty of evidence.

OUTCOME	STUDIES (PARTICIPANTS)	RISK OF BIAS	INDIRECTNESS	INCONSISTENCY	IMPRECISION	PUBLICATION BIAS	CERTAINTY OF EVIDENCE
Accuracy	33 (15,105)	Serious	Serious	Not serious	Serious	None	⨁◯◯◯ Very low
Sensitivity/Sensibility	5 (4,535)	Serious	Serious	Not serious	Serious	None	⨁◯◯◯ Very low
PPV	2 (1,508)	Serious	Serious	Not serious	Serious	None	⨁◯◯◯ Very low
AUC	3 (832)	Serious	Serious	Not serious	Serious	None	⨁◯◯◯ Very low
Other outcomes	5 (4,894)	Serious	Serious	Not serious	Serious	None	⨁◯◯◯ Very low

AUC: area under the curve; PPV: positive predictive value.

### Quality assessment of machine learning models

Box 4 summarizes the quality assessment data. The included articles presented adequate methodological attributes and limitations of the evaluated items. In total, 29 of the 48 (60.41%) articles showed consistent data of sample representativeness, but only four indicated the control of confounding variables (8.33%). All studies used machine learning algorithms and included model performance data (100%). A total of 45 studies consistently reported the assessment of outcomes (93.75%). Moreover, 18 articles addressed the handling of missing data (37.5%).

**Box 4:** Studies quality assessment.

STUDY	SAMPLE REPRESENTATIVENESS	CONTROL CONFOUNDING VARIABLES	ASSESSMENT OF THE OUTCOME	MACHINE LEARNING ALGORITHM	PERFORMANCE METRICS	MISSING DATA	TEST UNSEEN	CLASS IMBALANCE	FEATURE SELECTION + HYPERPARAMETER
Amalraj et al. ^30^ (2023)	No	No	No	Yes	Yes	Yes	Yes	No	No
Jiao et al. ^31^ (2023)	No	No	Yes	Yes	Yes	No	Yes	No	No
Pal et al. ^32^ (2023)	No	No	Yes	Yes	Yes	Yes	Yes	No	No
Pourmohammadi & Maleki ^33^ (2020)	No	No	Yes	Yes	Yes	No	Yes	No	Yes
Sharma et al. ^34^ (2022)	No	No	Yes	Yes	Yes	No	Yes	No	Yes
Silva et al. ^35^ (2020)	No	No	Yes	Yes	Yes	No	Yes	No	No
Tiwari & Agarwal ^36^ (2021)	No	No	Yes	Yes	Yes	No	Yes	Yes	Yes
Anand et al. ^37^ (2023)	Yes	No	Yes	Yes	Yes	No	Yes	Yes	No
Balli et al. ^38^ (2023)	No	No	Yes	Yes	Yes	No	No	No	No
Daza et al. ^39^ (2023)	Yes	No	Yes	Yes	Yes	Yes	Yes	Yes	No
Estabragh et al. ^40^ (2013)	Yes	No	Yes	Yes	Yes	No	No	No	No
Herbert et al. ^41^ (2021)	Yes	No	Yes	Yes	Yes	Yes	Yes	No	Yes
Ge et al. ^42^ (2020)	Yes	No	Yes	Yes	Yes	No	Yes	No	No
Gil et al. ^43^ (2022)	Yes	No	Yes	Yes	Yes	Yes	Yes	No	Yes
Maitre et al. ^44^ (2023)	Yes	No	Yes	Yes	Yes	No	Yes	No	No
Morales-Rodríguez et al. ^45^ (2021)	Yes	No	Yes	Yes	Yes	Yes	Yes	No	Yes
Ren et al. ^46^ (2021)	Yes	No	Yes	Yes	Yes	No	Yes	Yes	No
Upadhyay et al. ^47^ (2023)	Yes	No	Yes	Yes	Yes	No	Yes	No	No
Vergaray et al. ^48^ (2022)	Yes	No	Yes	Yes	Yes	Yes	Yes	Yes	No
Wang et al. ^49^ (2020)	Yes	No	Yes	Yes	Yes	No	Yes	No	No
AlShorman et al. ^50^ (2022)	Yes	No	Yes	Yes	Yes	No	Yes	No	No
He et al. ^51^ (2021)	Yes	Yes	Yes	Yes	Yes	Yes	Yes	No	Yes
Li et al. ^52^ (2015)	No	No	Yes	Yes	Yes	No	Yes	Yes	No
Modinos et al. ^53^ (2013)	No	Yes	Yes	Yes	Yes	No	Yes	No	No
Zhang et al. ^54^ (2019)	Yes	No	Yes	Yes	Yes	No	Yes	No	
Liu et al. ^55^ (2023)	Yes	Yes	Yes	Yes	Yes	No	Yes	No	No
Topalovic et al. ^56^ (2021)	No	No	Yes	Yes	Yes	No	No	No	No
Ding et al. ^57^ (2020)	Yes	No	Yes	Yes	Yes	No	Yes	No	No
Dehghan-Bonari et al. ^58^ (2023)	No	No	No	Yes	Yes	Yes	Yes	No	No
Siraji et al. ^59^ (2023)	Yes	No	No	Yes	Yes	No	Yes	No	Yes
Zhang et al. ^60^ (2020)	No	Yes	Yes	Yes	Yes	No	Yes	No	Yes
Ware et al. ^61^ (2020)	Yes	No	Yes	Yes	Yes	Yes	Yes	Yes	Yes
Aalbers et al. ^62^ (2023)	Yes	No	Yes	Yes	Yes	No	Yes	No	Yes
Acikmese & Alptekin ^63^ (2019)	No	No	Yes	Yes	Yes	No	Yes	Yes	No
Ahmed & Ahmed ^64^ (2023)	No	No	Yes	Yes	Yes	No	Yes	No	Yes
Chikersal et al. ^65^ (2021)	No	No	Yes	Yes	Yes	Yes	Yes	No	Yes
Guerrero et al. ^66^ (2023)	Yes	No	Yes	Yes	Yes	No	Yes	No	No
Mahalingam et al. ^67^ (2023)	Yes	No	Yes	Yes	Yes	Yes	No	No	Yes
Meda et al. ^68^ (2023)	Yes	No	Yes	Yes	Yes	No	Yes	No	Yes
Nemesure et al. ^69^ (2021)	Yes	No	Yes	Yes	Yes	Yes	Yes	No	Yes
Bhadra & Kumar ^70^ (2024)	Yes	No	Yes	Yes	Yes	Yes	Yes	No	Yes
Rois et al. ^71^ (2021)	Yes	No	Yes	Yes	Yes	No	Yes	No	Yes
Sano et al. ^72^ (2018)	Yes	No	Yes	Yes	Yes	Yes	Yes	Yes	Yes
Ware et al. ^73^ (2022)	No	No	Yes	Yes	Yes	No	Yes	No	No
Xu et al. ^74^ (2021)	Yes	No	Yes	Yes	Yes	Yes	Yes	No	No
Yue et al. ^75^ (2021)	No	No	Yes	Yes	Yes	Yes	Yes	No	Yes
Müller et al. ^76^ (2021)	No	No	Yes	Yes	Yes	No	Yes	No	Yes
Nayan et al. ^77^ (2022)	Yes	No	Yes	Yes	Yes	Yes	Yes	No	No

No: non-compliant with criteria; Yes: compliant with criteria.

Regarding the specific characteristics of machine learning models, 44 studies specified the sample split between testing and validation (91.66%). In total, nine articles addressed the resolution of the class imbalance issue (18.75%). Finally, 21 studies commented on feature selection from the dataset (43.75%).

## Discussion

The current systematic review aims to assess the performance of various machine learning models in predicting and detecting depression, anxiety, and stress in college students. A diverse range of models were examined among the 48 studies, including physiological, behavioral, internet usage, neurocerebral, blood markers, mixed, mobility, and demographic data. Overall, these machine learning models demonstrated satisfactory performance in predicting and classifying the intended outcomes.

Out of all the studies assessed, 33 of them ^30^ ^,^ ^31^ ^,^ ^32^ ^,^ ^33^ ^,^ ^34^ ^,^ ^36^ ^,^ ^37^ ^,^ ^38^ ^,^ ^39^ ^,^ ^42^ ^,^ ^43^ ^,^ ^46^ ^,^ ^47^ ^,^ ^48^ ^,^ ^49^ ^,^ ^50^ ^,^ ^51^ ^,^ ^52^ ^,^ ^53^ ^,^ ^54^ ^,^ ^56^ ^,^ ^57^ ^,^ ^58^ ^,^ ^59^ ^,^ ^63^ ^,^ ^64^ ^,^ ^65^ ^,^ ^67^ ^,^ ^70^ ^,^ ^71^ ^,^ ^72^ ^,^ ^74^ ^,^ ^77^ reported at least one accuracy score, whereas ten studies ^35^ ^,^ ^40^ ^,^ ^45^ ^,^ ^61^ ^,^ ^66^ ^,^ ^68^ ^,^ ^69^ ^,^ ^73^ ^,^ ^75^ ^,^ ^76^ relied solely on any metrics among F1, AUC, PPV, sensitivity, and specificity and five ^41^ ^,^ ^44^ ^,^ ^55^ ^,^ ^60^ ^,^ ^62^ studies presented other metrics, such as regression or correlation coefficients. All models exhibited at least one acceptable performance score, that is, above 0.5. Stress detection accuracy ranged from 63% to 100%, anxiety detection accuracy ranged from 53.68% to 97.9%, and depression detection accuracy ranged from 73.5% to 99.1%. These results raise the hypothesis that models targeting stress detection may exhibit subtly higher accuracy compared to those for anxiety and depression. However, further investigation with more homogeneous and comprehensive data is essential to test this hypothesis.

Regarding accuracy specifically, 30 out of these 33 studies (90.9%) ^30^ ^,^ ^31^ ^,^ ^32^ ^,^ ^33^ ^,^ ^34^ ^,^ ^36^ ^,^ ^37^ ^,^ ^38^ ^,^ ^39^ ^,^ ^42^ ^,^ ^43^ ^,^ ^46^ ^,^ ^47^ ^,^ ^48^ ^,^ ^49^ ^,^ ^50^ ^,^ ^52^ ^,^ ^53^ ^,^ ^54^ ^,^ ^56^ ^,^ ^57^ ^,^ ^58^ ^,^ ^59^ ^,^ ^64^ ^,^ ^65^ ^,^ ^70^ ^,^ ^71^ ^,^ ^72^ ^,^ ^74^ ^,^ ^77^ reported at least one accuracy score above 70%, categorizing them as achieving good accuracy ^23^. Additionally, 26 out of these 33 studies (78.78%) ^30^ ^,^ ^31^ ^,^ ^32^ ^,^ ^33^ ^,^ ^34^ ^,^ ^36^ ^,^ ^37^ ^,^ ^38^ ^,^ ^39^ ^,^ ^42^ ^,^ ^43^ ^,^ ^46^ ^,^ ^47^ ^,^ ^48^ ^,^ ^49^ ^,^ ^50^ ^,^ ^52^ ^,^ ^54^ ^,^ ^57^ ^,^ ^58^ ^,^ ^59^ ^,^ ^65^ ^,^ ^70^ ^,^ ^71^ ^,^ ^72^ ^,^ ^77^ achieved at least one accuracy score above 80%, which can be classified as excellent accuracy ^23^. These findings align with other systematic reviews in the field of mental health, which also identified satisfactory performance in most models that assessed conditions such as post-traumatic stress, depression, suicidal ideation, and anxiety ^21^ ^,^ ^22^ ^,^ ^23^ ^,^ ^24^ ^,^ ^25^ ^,^ ^78^. It is plausible that these models could exhibit enhanced accuracy by accounting for the influence of potential comorbidities, given that the presence of other psychopathological symptoms may impact the precision of machine learning models ^25^.

The studies that demonstrated the best model performance employed physiological data and showed stress as an outcome. Pourmohammadi & Maleki ^33^ and Tiwari & Agarwal ^36^ developed models with accuracies of 100% and 99.4%, respectively. A possible explanation is that machine learning models based on data correlated with the outcome tend to perform better ^25^. The association between stress variables and parameters such as blood pressure, skin conductivity, and heart rate are well-established and can account for these positive results ^79^. However, we highlight that both studies induced stress via a laboratory experiment, which differs from the stress experienced in an academic context.

Conversely, the two studies ^51^ ^,^ ^69^ that exhibited the lowest models performances were based on neuroimaging ^51^ and mixed data ^69^. He et al. ^51^ found a specificity of 32.88% in distinguishing individuals with anxiety from those with symptoms of schizophrenia. This observation can be partially attributed to the linear relationship between anxiety and psychosis variables, possibly implicating the activation of overlapping brain regions ^51^. On the other hand, Nemesure et al. ^69^ reported a sensitivity of merely 55% in identifying major depression among university students.

When examining the performance of machine learning algorithms, it is not possible to definitively assert the superiority of any specific technique. Algorithmic performance is contingent upon specific factors, including objectives, data volume and type, case distribution, outlier, noise management, among others. Consequently, the presence of a diverse array of algorithms in the evaluated studies is expected, given the variations in objectives, data types, and dataset characteristics. In this systematic review, SVM algorithms and their variations predominate, accounting for 35.41% of cases, a trend also observed in other systematic reviews within the field of mental health ^23^ ^,^ ^25^. This could be attributed to the fact that SVM algorithms excel in processing structured data, particularly in binary outcome classifications.

If, on the one hand, the performance data is promising, on the other hand, it is important to highlight that only one study ^51^ indicated external validation of the machine learning model. machine learning models that only rely on internal validation may overestimate their performance. Further studies must perform external validation of their machine learning models to disseminate them among the population.

Despite the adequate results, it should be noted that the quality of evidence from all studies was considered very low after GRADE assessment. These results suggest the importance of conducting studies that improve the assessment of outcomes and use larger and more representative samples. Issues pertaining to the construction of machine learning models were also identified. Only nine ^35^ ^,^ ^37^ ^,^ ^39^ ^,^ ^46^ ^,^ ^48^ ^,^ ^52^ ^,^ ^61^ ^,^ ^63^ ^,^ ^72^ studies outlined measures to address class imbalance. Moreover, several studies featured a sample size of fewer than 55 participants, which is considered small ^80^.

Most models may inherit limitations from the diagnostic process itself. Psychometric instruments, such as the *Beck Depression Inventory-II*, inherently possess measurement errors that can be replicated in these models. Moreover, these instruments can be influenced by respondents’ tendencies towards socially desirable responses. It is essential that mental health diagnoses stem from a triangulation of diverse sources of evidence ^80^, including qualitative and exploratory data, clinical interviews, observational data, and self-report instruments. Notably, only seven studies ^47^ ^,^ ^54^ ^,^ ^61^ ^,^ ^69^ ^,^ ^70^ ^,^ ^73^ ^,^ ^75^ constructed models after evaluation by healthcare professionals. However, the literature points that involving trained clinicians in this process can be more resource-intensive ^80^.

The findings of this systematic review offer promise from a public health perspective, indicating that machine learning algorithms may serve as valuable tools for the detection of depression, anxiety, and stress among university students using various types of data. Consequently, they show potential to enhance mental health support for university students, particularly those in remote or rural areas. These algorithms can aid identifying students at risk or flagging cases of depression, anxiety, and stress. Moreover, this study aligns with previous research endorsing the application of machine learning in mental healthcare ^81^. Although some machine learning initiatives have been under development in other regions, we highlight that most assessed studies are concentrated in European countries, China, and the United States. Expanding machine learning research and implementation in developing countries could significantly contribute to the advancement of mental healthcare worldwide.

Finally, a potential challenge to the widespread adoption of machine learning models in public health is the type of data they depend on. Models that rely on neuroimaging and physiological data collected via electromyograms and electroencephalograms demand specialized data collection and can present practical challenges for real-world implementation ^25^. In contrast, models that are built employing behavioral data gathered from research or even linguistic interactions on social networks may offer a more practical and feasible approach. Therefore, it is crucial to assess the potential challenges and advantages associated with each model when applied to real-world contexts. Additionally, the consideration of ethical issues is of significance.

### Ethical issues

The use of machine learning has sparked ethical discussions, particularly regarding the privacy of personal data and the purpose of these models. Interestingly, only a few studies ^40^ ^,^ ^41^ in this review mentioned ethical issues related to machine learning. Models should prioritize the protection of personal data, especially when dealing with sensitive content, such as language patterns and interactions on social networks, as well as smartphones messages and calls. Moreover, these algorithms must solely aim at identifying mental health issues for prevention and promotion of mental well-being, protecting sensitive data from vested interests.

### Limitations

This systematic review shows methodological limitations that suggest caution in interpreting and generalizing the results. These limitations refer to the inclusion and exclusion criteria and quality of the machine learning models.

Firstly, the inclusion criteria may have limited the number of articles. To refine the quality of the articles, we decided to exclude those published in gray literature. Thus, book chapters and articles from conference proceedings and references were excluded. Secondly, based on previous research, we found no validated instrument to assess the quality of machine learning articles. Therefore, an instrument that has not yet been validated ^23^ was employed to assess the articles.

## Conclusion

The findings of this review suggest that most machine learning models demonstrate adequate performance in assessing the intended outcomes, particularly stress. Various types of data were employed in these machine learning models, indicating that depression, anxiety, and stress may be predicted or classified using various approaches, although concerns persist regarding the certainty of evidence of models, which may be considered very low. These results hold promise for the application of machine learning in public health, as it can assist in identifying students at risk of mental illness or those experiencing depression, anxiety, and stress.

Machine learning algorithms show the potential to significantly enhance the accessibility of mental health services by enabling accurate real-time assessments, often remotely, even with non-linear data. This capacity is especially valuable for improving mental healthcare in rural or underserved areas with limited access to traditional mental health services. Thus, we suggest further development of machine learning models, with a particular focus on incorporating various sources of evidence for classifying outcomes, beyond solely relying on self-report instruments. It is essential that future studies also perform external validation of machine learning models to obtain more consistent and realistic performance data. Wider dissemination of these studies can facilitate the adoption of more rigorous statistical techniques, including meta-analysis, which can offer more conclusive insights into the performance and practical utility of these models.
