# Supplementary material for: How do machine learning models perform in the detection of depression, anxiety, and stress among undergraduate students? A systematic review
Source: Cad Saude Publica. 2024 Dec 20;40(11):e00029323. doi: 10.1590/0102-311XEN029323 (PMC11654111; doi:10.1590/0102-311XEN029323)
Supplement: Supplementary file 1 [file 1678-4464-csp-40-11-EN029323-s.pdf]

SUPPLEMENTARY MATERIAL

Box S1 Complete search strategy.

DATABASE	COMPLETE SEARCH STRATEGY
PubMed	(Depression[mh] OR Depressive disorder[mh] OR depress*[tiab] OR Anxiety[mh] OR Anxiety Disorders[mh] OR anxiet*[tiab] OR angst[tiab] OR anxious*[tiab] OR hypervigilance[tiab] OR nervousness[tiab] OR Stress, Psychological[mh] OR stress[tiab] OR "psychological stress*"[tiab] OR Mental Health[mh] OR Mental Health[tiab])<br>AND<br>(Machine Learning[mh] OR machine learning[tiab] OR transfer learning[tiab] OR artificial intelligence[tiab] OR supervised learning[tiab] OR unsupervised learning[tiab] OR big data[tiab] OR machine intelligence[tiab])<br>AND<br>(Students[mh] OR Universities[mh] OR Universit*[tiab] OR Colleg*[tiab] OR Undergraduat*[tiab] OR "Higher Education"[tiab] OR academic*[tiab] OR scholastic*[tiab])
PsycINFO	((IndexTermsFilt: ("College Students") OR IndexTermsFilt: ("Junior College Students") OR IndexTermsFilt: ("Community College Students") OR IndexTermsFilt: ("International Students") OR IndexTermsFilt: ("Colleges") OR IndexTermsFilt: ("Community Colleges") OR IndexTermsFilt: ("Higher Education") OR IndexTermsFilt: ("Undergraduate Education")) OR (title: (student*) OR title: (academic*) OR title: (scholastic*) OR title: (Universit*) OR title: (Colleg*) OR title: (Undergraduat*) OR title: ("Higher Education")) OR (abstract: (student*) OR abstract: (academic*) OR abstract: (scholastic*) OR abstract: (Universit*) OR abstract: (Colleg*) OR abstract: (Undergraduat*) OR abstract: ("Higher Education")) OR (Keywords: (student*) OR Keywords: (academic*) OR Keywords: (scholastic*) OR Keywords: (Universit*) OR Keywords: (Colleg*) OR Keywords: (Undergraduat*) OR Keywords: ("Higher Education"))) AND ((IndexTermsFilt: ("Machine Learning") OR IndexTermsFilt: ("Artificial Neural Networks") OR IndexTermsFilt: ("Computational Reinforcement Learning") OR IndexTermsFilt: ("Generative Artificial Intelligence") OR IndexTermsFilt: ("Inductive Logic Programming") OR IndexTermsFilt: ("Machine Learning Algorithms") OR IndexTermsFilt: ("Predictive Analysis") OR IndexTermsFilt: ("Supervised Learning") OR IndexTermsFilt: ("Unsupervised Learning") OR IndexTermsFilt: ("Big Data")) OR (title: ("machine learning") OR title: ("transfer learning") OR title: ("artificial intelligence") OR title: ("supervised learning") OR title: ("unsupervised learning") OR title: ("big data") OR title: ("machine intelligence")) OR (abstract: ("machine learning") OR abstract: ("transfer learning") OR abstract: ("artificial intelligence") OR abstract: ("supervised learning") OR abstract: ("unsupervised learning") OR abstract: ("big data") OR abstract: ("machine intelligence")) OR (Keywords: ("machine learning") OR Keywords: ("transfer learning") OR Keywords: ("artificial intelligence") OR Keywords: ("supervised learning") OR Keywords: ("unsupervised learning") OR Keywords: ("big data") OR Keywords: ("machine intelligence"))) AND ((IndexTermsFilt: ("Depression (Emotion)") OR IndexTermsFilt: ("Anaclitic Depression") OR IndexTermsFilt: ("Dysthymic Disorder") OR IndexTermsFilt: ("Endogenous Depression") OR IndexTermsFilt: ("Reactive Depression") OR IndexTermsFilt: ("Recurrent Depression") OR IndexTermsFilt: ("Treatment Resistant Depression") OR IndexTermsFilt: ("Atypical Depression") OR IndexTermsFilt: ("Persistent Depressive Disorder") OR IndexTermsFilt: ("Major Depression") OR IndexTermsFilt: ("Anxiety") OR IndexTermsFilt: ("Computer Anxiety") OR IndexTermsFilt: ("Death Anxiety") OR IndexTermsFilt: ("Health Anxiety") OR IndexTermsFilt: ("Mathematics Anxiety") OR IndexTermsFilt: ("Performance Anxiety") OR IndexTermsFilt: ("Social Anxiety") OR IndexTermsFilt: ("Speech Anxiety") OR IndexTermsFilt: ("Test Anxiety") OR IndexTermsFilt: ("Anxiety Disorders") OR IndexTermsFilt: ("Generalized Anxiety Disorder") OR IndexTermsFilt: ("Stress") OR IndexTermsFilt: ("Academic Stress") OR IndexTermsFilt: ("Social Stress") OR IndexTermsFilt: ("Acute Stress") OR IndexTermsFilt: ("Distress") OR IndexTermsFilt: ("Chronic Stress") OR IndexTermsFilt: ("Psychological Stress") OR IndexTermsFilt: ("Youth Mental Health") OR IndexTermsFilt: ("Mental Health") OR IndexTermsFilt: ("Emotional Health")) OR (title: (depress*) OR title: (anxiet*) OR title: (angst) OR title: (anxious*) OR title: (hypervigilance) OR title: (nervousness) OR title: (stress) OR title: ("psychological stress*") OR title: ("Mental health")) OR (abstract: (depress*) OR abstract: (anxiet*) OR abstract: (angst) OR abstract: (anxious*) OR abstract: (hypervigilance) OR abstract: (nervousness) OR abstract: (stress) OR abstract: ("psychological stress*") OR abstract: ("Mental health")) OR (Keywords: (depress*) OR Keywords: (anxiet*) OR Keywords: (angst) OR Keywords: (anxious*) OR Keywords: (hypervigilance) OR Keywords: (nervousness) OR Keywords: (stress) OR Keywords: ("psychological stress*") OR Keywords: ("Mental health")))
Web of Science	TS=(depress* OR anxiet* OR angst OR anxious* OR hypervigilance OR nervousness OR stress OR "psychological stress*" OR "Mental health")<br>AND<br>TS=("machine learning" OR "transfer learning" OR "artificial intelligence" OR "supervised learning" OR "unsupervised learning" OR "big data" OR "machine intelligence")<br>AND<br>TS=(student* OR academic* OR scholastic* OR Universit* OR Colleg* OR Undergraduat* OR "Higher Education")
Embase	('depression'/exp OR 'anxiety'/exp OR 'anxiety disorder'/exp OR 'mental stress'/exp OR 'mental health'/exp OR (depress* OR anxiet* OR angst OR anxious* OR hypervigilance OR nervousness OR stress OR 'psychological stress*' OR 'mental health'):ti,ab,kw)<br>AND<br>('machine learning'/exp OR ('machine learning' OR 'transfer learning' OR 'artificial intelligence' OR 'supervised learning' OR 'unsupervised learning' OR 'big data' OR 'machine intelligence'):ti,ab,kw)<br>AND<br>('college student'/exp OR 'health student'/exp OR 'undergraduate student'/exp OR 'university student'/exp OR 'college'/exp OR 'community college'/exp OR 'medical school'/exp OR 'university'/exp OR (student* OR academic* OR scholastic* OR Universit* OR Colleg* OR Undergraduat* OR "Higher Education"):ti,ab,kw)<br>AND<br>[embase]/lim NOT ([embase]/lim AND [medline]/lim)

Box S2 References excluded after full reading.

REFERENCES EXCLUDED AFTER FULL READING	REASON FOR EXCLUSION
Saha K, Yousuf A, Boyd RL, Pennebaker JW, De Choudhury M. Social Media Discussions Predict Mental Health Consultations on College Campuses. Sci Rep 2022; 12(1):123.	Did not present depression, anxiety or stress as an outcome.
Mei G, Xu W, Li L, Zhao Z, Li H, Liu W, et al. The Role of Campus Data in Representing Depression Among College Students: Exploratory Research. JMIR Ment Health 2020; 7:e12503.	Did not use machine learning.
Orozco-del-Castillo MG, Orozco-del-Castillo E, Brito-Borges E, Bermejo-Sabbagh C, Cuevas-Cuevas N. An artificial neural network for depression screening and questionnaire refinement in undergraduate students. International Congress of Telematics and Computing; 2021; Cham: Springer International Publishing.	Grey literature.
Iliou T, Konstantopoulou G, Stephanakis I, Anastasopoulos K, Lymberopoulos D, Anastassopoulos G. Iliou machine learning data preprocessing method for stress level prediction. Proceedings of 14th IFIP WG 12.5 International Conference; 2018 May 25–27; Rhodes, Greece.	Grey literature.
Yan J, Li XY, Geng YL, Liang YF, Chen C, Han ZW, et al. A preliminary prediction model of depression based on whole blood cell count by machine learning method. Zhonghua yu Fang yi xue za zhi [Chinese Journal of Preventive Medicine] 2023; 57(11):1862-1868.	Other language (article written in Mandarin language).
Jones BW, Taylor WD, Walsh CG. Sequential autoencoders for feature engineering and pretraining in major depressive disorder risk prediction. JAMIA 2023; 6(4):ooad086.	Other public.
Baba A, Bunji K. Prediction of mental health problem using annual student health survey: machine learning approach. JMIR Ment Health 2023; 10:e42420.	Did not present depression, anxiety or stress as an outcome (article does not specify the assessment of depression, anxiety or stress outcomes).
Park CH, Kwon J, Lee JT, Ahn S. Impact of Criterion Versus Norm-Referenced Assessment on the Quality of Life in Korean Medical Students. J Korean Med Sci. 2023; 38(17):e133.	Did not use machine learning (machine learning used differently).
Benchekroun M, Velmovitsky PE, Istrate D, Zalc V, Morita PP, Lenne, D. Cross dataset analysis for generalizability of HRV-based stress detection models. Sensors 2023; 23(4):1807.	Other public.
Sanchez-Trigo H, Molina-Martínez E, Grimaldi-Puyana M, Sañudo B. Effects of lifestyle behaviours and depressed mood on sleep quality in young adults. A machine learning approach. Psychol Health 2024; 39(1):128-143.	Did not present depression, anxiety or stress as an outcome.
Chuinsiri N. Unsupervised Machine Learning Identified Distinct Population Clusters Based on Symptoms of Oral Pain, Psychological Distress, and Sleep Problems. J Int Soc Prev Community Dent 2021; 11(5):531-538.	Did not present depression, anxiety or stress as an outcome (article does not specify the assessment of depression, anxiety or stress outcomes).
Tyagi S, Mehta A, Gupta V. Prediction of Stress and Causing factors using Machine learning: An Experimental Analysis. NeuroQuantology 2022; 20(7):1821:1827.	Other public.
Vanitha V, Krishnan P. Real time stress detection system based on EEG signals. Biomedical Research 2017; S-271-S274.	Grey literature.
Kvtkn P, Ramakrishnudu T. A novel method for detecting psychological stress at tweet level using neighborhood tweets. J King Saud Univ Comput Inf Sci 2022; 34(9):6663-6680.	Other public.
Rahman AA, Siraji MI, Khalid LI, Faisal F, Nishat MM, Ahmed A, et al. Perceived stress analysis of undergraduate students during covid-19: a machine learning approach. Proceedings of IEEE 21st Mediterranean Electrotechnical Conference (MELECON); 2022.	Grey literature.
Alsuwailem RI, Bhatia S. Detection and Mathematical Modeling of Anxiety Disorder Based on Socioeconomic Factors Using Machine Learning Techniques. Human-centric Computing and Information Sciences 2022; 12(52):1-17.	Other public (the dataset covers college students and non-student participants).
Xu H, Wu X, Liu X. A measurement method for mental health based on dynamic multimodal feature recognition. Front Public Health 2022; 10:990235.	Did not present depression, anxiety or stress as an outcome (article does not specify the assessment of depression, anxiety or stress outcomes).
Li C, Yang M, Zhang Y, Lai KW. An Intelligent Mental Health Identification Method for College Students: A Mixed-Method Study. Int J Environ Res Public Health 2022;19(22):14976.	Did not present depression, anxiety or stress as an outcome (article assess emotion recognition).
Ya'kob NAA, Farook RSM, Halim AHA, Fadzil MFM, Rejab MRA, Elias SJ. Motion Detection System for Recognition of Early Sign of Depression. Proceedings of 6th IEEE International Conference on Recent Advances and Innovations in Engineering (ICRAIE); 2021.	Grey literature.
Miranda-Piña G, Alejo R, Rendón E, Granda-Gutíerrez EE, Valdovinos RM, del Razo-López F. Clustering Analysis in the Student Academic Activities on COVID-19	Grey literature.

Pandemic in Mexico. Proceedings of International Conference on Intelligent Computing; 2019; Cham: Springer International Publishing; 2019.	
Liu N, Liu H, Liu H. Mental health diagnosis of college students based on facial recognition and neural network. Journal of Intelligent & Fuzzy Systems 2021; 40(4):7061-7072.	Did not present depression, anxiety or stress as an outcome (article does not specify the assessment of depression, anxiety or stress outcomes).
Choudhury AA, Khan MRH, Nahim NZ, Tulon SR, Islam S, Chakrabarty A. Predicting depression in Bangladeshi undergraduates using machine learning; Proceedings of IEEE Region 10 Symposium; 2019.	Grey literature.
Yanto ITR, Vitasari P, Herawan T, Deris MM. Applying variable precision rough set model for clustering student suffering study's anxiety. Expert Syst Appl 2012; 39(1):452-459.	Did not present depression, anxiety or stress as an outcome. (anxiety in academic tasks).
Ruiz FAA, Bustamante DAG, González KO, Sandoval KM. Árbol de clasificación para la identificación de síntomas asociados a la depresión en estudiantes de una universidad pública. Retos 2024; (52):104-114.	Other language (article written in Spanish language).
Aguilera A, Figueroa CA, Hernandez-Ramos R, Sarkar U, Cemballi A, Gomez-Pathak L, et al. mHealth app using machine learning to increase physical activity in diabetes and depression: clinical trial protocol for the DIAMANTE Study. BMJ Open 2020; 10(8):e034723.	Did not present depression, anxiety or stress as an outcome (article assesses diabetes).
Zhao FF. Artificial neural network application for identifying risk of depression in high school students: a cross-sectional study. BMC Psychiatry 2021; 21(1):517.	Other public (high school students).
Nuankaew WS, Nasa-Ngium P, Enkvetchakul P, Nuankaew P. A Predictive Model for Depression Risk in Thai Youth during COVID-19. J Adv Inf Technol 2022; 13(5):450-455.	Other public (high school students along with college students).
Benfares C, Akhrif O, El Idrissi YEB, Hamid K. A clinical support system for classification and prediction of depression using machine learning methods. Comput Intell 2021; 37(4): 1619-1632.	Other public (patients from hospital).
Pei J. Prediction and Analysis of Contemporary College Students' Mental Health Based on Neural Network. Comput Intell Neurosci 2022; 2022:7284197	Did not present depression, anxiety or stress as an outcome (article does not specify the assessment of depression, anxiety or stress outcomes).
Sun S, Dong Y, Li Y, Liu H. A mechanism for analyzing and managing undergraduates' mental health based on large-scale behavior data: AI-based approach. Internet Technol Lett 2023; e467.	Did not present depression, anxiety or stress as an outcome. (article does not specify the assessment of depression, anxiety or stress outcomes).
Chen T. Investigating the mental health of university students during the COVID-19 pandemic in a UK university: a machine learning approach using feature permutation importance. Brain Inform 2023; 10(1):27.	Did not use machine learning (machine learning used differently).
Al-Wesabi FN, Alsolai H, Hilal AM, Hamza M, Al Duhayyim M, Negm N. Machine learning based depression, anxiety, and stress predictive model during COVID-19 crisis. Comput Mater Contin 2022; 70(3):5803-5820.	Grey literature.
Rekha KSS, Mathur S, Sadhukhan S, Jangiti J. Student Stress Prediction Using Machine Learning Algorithms and Comprehensive Analysis. NeuroQuantology 2022; 20(14): 895-906.	Grey literature.
Mahayossanunt Y, Nupairoj N, Hemrungrojn S, Vateekul P. Explainable Depression Detection Based on Facial Expression Using LSTM on Attentional Intermediate Feature Fusion with Label Smoothing. Sensors 2023; 23(23):9402.	Did not use machine learning (article uses deep learning).
Ratul IJ, Nishat MM, Faisal F, Sultana S, Ahmed A, Al Mamun MA. Analyzing Perceived Psychological and Social Stress of University Students: A Machine Learning Approach. Heliyon 2023; 9(6):e17307.	Other public (undergraduate students along with postgraduate students).
Qirtas MM, Zafeiridi E, White EB, Pesch D. The relationship between loneliness and depression among college students: Mining data derived from passive sensing. Digit Health 2023; 9:20552076231211104.	Other public (undergraduate students along with postgraduate students).
Talukder A, Hasan MM, Haq I, Shariful SM. A machine learning model for the identification of depressive symptoms among university students in Bangladesh. Minerva Psichiatr 2022; 63(3):237-244	Other public (undergraduate students along with postgraduate students).
